# Nanolasers with Feedback as Low-Coherence Illumination Sources for Speckle-Free Imaging: A Numerical Analysis of the Superthermal Emission Regime

**DOI:** 10.3390/nano11123325

**Published:** 2021-12-07

**Authors:** Tao Wang, Can Jiang, Junlong Zou, Jie Yang, Kuiwen Xu, Chaoyuan Jin, Gaofeng Wang, Gian Piero Puccioni, Gian Luca Lippi

**Affiliations:** 1 School of Electronics and Information, Hangzhou Dianzi University, Hangzhou 310018, China; jiangcan@hdu.edu.cn (C.J.); yanglime@hdu.edu.cn (J.Y.); kuiwenxu@hdu.edu.cn (K.X.); 2 School of Communication Engineering, Hangzhou Dianzi University, Hangzhou 310018, China; zjl418597114@hdu.edu.cn; 3 College of Information Science and Electronic Engineering, Zhejiang University, Hangzhou 310007, China; jincy@zju.edu.cn; 4 International Joint Innovation Center, Zhejiang University, Haining 314400, China; 5 Zhejiang Laboratory, Hangzhou 311121, China; 6 Istituto dei Sistemi Complessi, CNR, 50019 Sesto Fiorentino, Italy; gianpiero.puccioni@isc.cnr.it; 7 Institut de Physique de Nice (INPHYNI), Université Côte d’Azur, 06103 Nice, France

**Keywords:** semiconductor nanolaser, superthermal emission, speckle reduction, self-feedback, low-coherence

## Abstract

Lasers distinguish themselves for the high coherence and high brightness of their radiation, features which have been exploited both in fundamental research and a broad range of technologies. However, emerging applications in the field of imaging, which can benefit from brightness, directionality and efficiency, are impaired by the speckle noise superimposed onto the picture by the interference of coherent scattered fields. We contribute a novel approach to the longstanding efforts in speckle noise reduction by exploiting a new emission regime typical of nanolasers, where low-coherence laser pulses are spontaneously emitted below the laser threshold. Exploring the dynamic properties of this kind of emission in the presence of optical reinjection we show, through the numerical analysis of a fully stochastic approach, that it is possible to tailor some of the properties of the emitted radiation, in addition to exploiting this naturally existing regime. This investigation, therefore, proposes semiconductor nanolasers as potential attractive, miniaturized and versatile future sources of low-coherence radiation for imaging.

## 1. Introduction

Since the first demonstration by Maiman in 1960, lasers have become indispensable light sources that enable a wide range of consumer technologies and data communication systems while also promoting fundamental research in different fields [1]. Technological applications have also driven the search for miniaturization [2,3,4,5], which has attained, with advanced nanotechnology and nanofabrication, ultra-compact dimensions [6]. Initiated with the Vertical-Cavity Surface-Emitting Laser (VCSEL) [7] and passing through microdisks lasers (e.g., in whispering gallery configurations [8]), photonic crystal lasers [9] and plasmonic nanolasers [4,10,11,12], the cavity volume has shrunk to subwavelength size. Thanks to the limited number of optical modes in such small cavities, the spontaneous emission coupling factor β (fraction of spontaneous emission coupled into the lasing mode) become non-negligible, allowing for the amplification by stimulated emission at lower pumps; thus, reducing the laser threshold [13]. The emerging small-footprint, ultralow threshold nanolasers now enable a wide variety of applications in different fields [14,15,16,17].

Laser imaging, among them, has been attracting a great deal of attention thanks to the high brightness of its radiation, strong directionality, excellent color purity and high efficiency [18]. One additional advantage is the longer lifetime, normally one order of magnitude longer than that of arc lamps, while maintaining good properties throughout its operation [19]. It is, therefore, only natural that lasers should have attracted attention in the realm of imaging. However, the typical benefit offered by laser light—high spatial and temporal coherence [20]—does become a shortcoming when illumination is concerned since it leads to coherence artifacts and cross-talk in full-field imaging and display [21]: speckle noise is the most common manifestation of such an artifact, stemming from random interference patterns, which result from the coherent addition of scattered photons. Speckles are indeed a longstanding issue that reduce spatial resolution and image quality [22]; thus, hindering the use of lasers in full-field imaging applications [23].

Over the decades, different technologies have been developed for speckle-free laser illumination, with most strategies based on the reduction of spatial and temporal coherence of the laser emission [24,25,26], after early attempts at using Light-Emitting Diodes (LEDs) proved their strong power limitations [27]. Alternative techniques chose to circumvent the laser coherence through raster-scanning modalities [20], but the intrinsically slow scanning speeds strongly limited the temporal response, thus the system’s efficiency.

In order to reduce or eliminate speckle noise, different technologies have been developed for speckle-free uniform illumination, and most strategies are based on the reduction of spatial and temporal coherence of laser emission [24,25,26]. Lamps and LEDs were the initial choices, with the unfortunate accompanying power limitations [27]. Alternatives based on raster-scanning modalities in laser-based imaging systems [20] turned out to be too time-consuming, thus not sufficiently efficient. New cavity geometries [28,29], optical feedback, other dynamics [25,30,31,32] and random lasers and supercontinuum sources [20] were studied as low-coherence sources. Recently, an experimental investigation reported on the speckle patterns obtained from modulated microlasers and coupled-cavity nanolasers, where unexpected non-Rayleigh statistics could be related to ultrafast temporal fluctuations [33]. There the authors examine, in parallel, techniques to reduce emission coherence in an edge-emitting semiconductor laser and in the coupled nanolasers. Notice that an edge-emitter has a sufficiently large cavity volume (to simplify the image) to be considered a macroscopic laser, in spite of its reduced physical size [34,35,36]. This interesting parallel highlights the intrinsic differences between the two kinds of sources and the potential that nanodevices hold over macroscopic ones, as long as the photon flux delivers sufficient illumination.

Following this promising experimental demonstration, we focus here on a simpler system: a single nanolaser operated in the low-coherence emission regime, which naturally precedes the laser threshold. The interest resides in characterizing the potential usefulness of this intrinsic emission region, normally considered a shortcoming of ultra-small devices, to exploit its features at virtually no cost! Through simulations based on stochastic modeling of the photon emission, we numerically investigate the dynamic properties of their light in the lasing transition (threshold) region. In particular, we examine the possibility of exploiting the low coherence of the photon bursts that appear (as a form of Amplified Spontaneous Emission—ASE), with superthermal statistics ($g^{\left(2\right)}\left(0\right)>2$): proof of a dynamics consisting of independent photon spikes. Adding one external degree of freedom—optical feedback—we study the degree of control that can be gained on the emission properties. The aim is to study application-oriented, low-coherence light from sources possessing a very high efficiency, an extremely low thermal load and are so small as to be integrated on-chip or packaged close to fiber tips.

## 2. Experimental Implementability and Practical Interest

In order to motivate the investigation, we are going to examine a couple of possible implementations to evaluate their degree of interest and their potential impact. Our somewhat arbitrary choices are made for the sake of example, knowing that in a short time technological progress will enable even better schemes.

The first realization that can be envisaged is based on photonic crystal cavity nanolasers, electrically pumped and coupled to a waveguide, as in [37]. This choice allows us to examine some important properties of feedback into a nanolaser, under realistic conditions and with features that match those of actual devices.

Nanolaser output, in this configuration, is coupled into an underlying waveguide through suitable mode leakage and in an amount that is controlled during manufacturing. The symmetry of the configuration allows for the insertion of feedback at one end of the waveguide, which can be coupled to an optical fiber. The fiber length and termination (as well as fiber properties, such as added nanoscatterers [38]) determine the properties of the feedback arm: time delay and amplitude of the backscattered component. The other waveguide end couples the light out of the package, allowing for the inclusion of either coupling to another fiber to guide the light to the target or of matching optics to obtain a beam spot of the desired size on the target. Even though this ensemble of functionalities is not part of a standardized routine, all the steps are well mastered and can be counted upon to obtain functional components.

As shown in Figure S1, the amount of power to be expected in the nanolaser output– for the emission regime, which concerns this paper—is in the μWatt range. While such power levels provide only a very low level of lighting, the compactness of the source opens the way for a niche of applications where low photon fluxes are more than adequate and even particularly suitable. Medical probes are the first kind of devices where the performance of a low-coherence nanolaser may be envisaged, thanks to the close proximity of the environment and often the need for ultralow light levels (e.g., in ophthalmology). The extremely reduced footprint, together with very low power consumption and favorable thermal load, render a nanolaser-based illumination source integrable directly into probes. The laser’s feedback arm, in the form of a length of fiber, could be integrated into the bundle cord that supplies power and retrieves information, enabling the construction of a compact and flexible system.

A second realization targets stronger lighting levels. In Section 5.1, we compare the nanolaser emission to that of an equivalent-sized LED to show that its output is at least twice as intense; thus, offering better efficiency. LEDs are ideally suited as low-coherence illumination sources, being readily available and quite powerful. As such, they easily surpass the current expectations of nanolasers. However, it is not impossible to envisage future developments of nanolaser arrays operating in a low-coherence regime with a construction geometry similar to what is used for pumping solid-state lasers with diodes. The micropillar nanolaser configuration (based on VCSEL geometry) would be particularly suited to this task. Its advantages, in addition to superior individual source efficiency, could be summarized as follows:

1. Different micropillar nanolasers emit independently of one another, resulting in a lack of mutual coherence; thus, immediately improving on the (low) coherence properties expected from the source.
2. The useful illumination results from the direct sum of the power output by each source.
3. Collective feedback could reinject light into the array through reflective (or refractive) optics, mixing the photons emitted by the individual sources [39]; thus, further lowering the coherence of the array’s output.
4. Diffractive optics, engraved onto the top surface of the array, could be used to control the overall beam divergence, tailoring to the needs of the envisaged application [40].

Obtaining uniform features across the nanolaser matrix, ensuring the same operating regime, is a challenge, but similar issues are already being addressed for neuromorphic computing [41], where locking capabilities impose more stringent constraints than those imposed by lighting. Arrays with 100 × 100 elements could provide illumination in the range of 10 mW, offering alternative, compact sources whose tunable properties differ from those of LEDs. As such, they would not necessarily position themselves as LED replacements but rather as sources with complementary properties.

## 3. Numerical Simulations Based on a Fully Stochastic Method

In order to properly account for the statistical properties of the emission in the transition region, characterized by photon bursts [42,43], we make use of a Stochastic Simulator [44] (or Stochastic Laser Simulator, SLS), based on a semiclassical description [45] of lasing. The intrinsic advantage of the SLS is the rapid prediction of trajectories, and their statistics, without any hypotheses on the noise structure: all physical processes (including spontaneous emission, relaxations and photon transmission and reinjection) are modeled as probabilistic processes based on their characteristic time constants. This approach permits a high-quality reproduction of the physical properties of a laser without the difficulties that are inherent in the simulation of added noise sources [46,47]. It is important to stress that the discrete description of the interaction between photons and emitters—intrinsically discrete since only integer quanta can be exchanged—inherently contains all the noise sources without any need for additional assumptions while reproducing analytical noise features, which are much more difficult to obtain from numerical simulations with Langevin noise sources [47].

The numerical scheme is the same as the one implemented in [48]; therefore, we refer the reader to that publication for all details. For this investigation, we set the delay length to 60 cm, corresponding to 4 ns time delay. This choice represents a compromise between a sufficiently long delay to simulate interesting incoherent feedback (when the dynamics do not allow for continuous oscillation) and a short enough one to avoid extremely lengthy computations. Towards the end of the manuscript, we will discuss the extrapolation to different delay lengths and their usefulness.

All pump values are normalized by the so-called threshold pump $P_{th}$ expressed by $P_{th}=\frac{\Gamma_{c}}{\beta }$ [34]. This pump value, which corresponds to the midpoint in the steep portion of the steady-state curve representing photon number vs. pump [48], should not be confused with the true laser threshold, which takes place at larger pump values for a nanolaser. The feedback fraction is the ratio between the photons injected into the laser and the total output photons, defined by

(1) $$\begin{array}{c} \eta =\frac{S_{inj}}{S_{out}}\;, \end{array}$$

where $S_{inj}$ denotes the photon number injected into the laser, and $S_{out}$ represents the photon number outcoupled from the cavity. We explicitly introduce this parameter to mimic experimental setups where the reflectivity of one mirror is fixed, but the feedback tuning can be achieved through an additional element (e.g., an optical filter).

An intrinsic property of the SLS is its reliance on the radiation-matter interaction based on photon numbers. As such, it is not capable of providing direct spectral (or coherence) information, a shortcoming around which we need to develop an investigation strategy to answer questions related to low-coherence emission. Making up for this indubitable drawback, the SLS is the only existing laser model capable of predicting the (observed) pre-threshold dynamics. Thus, it is at the present time irreplaceable, in spite of the incomplete information that it can provide.

## 4. Investigation Strategy

Although the SLS model does not provide any information about the optical spectrum or the electric field, we can infer some of the coherence properties with the help of photon statistics. The statistical properties of laser emission can be characterized through the second-order correlation function [49]

(2) $$\begin{array}{c} g^{\left(2\right)}\left(\tau \right)=\frac{\langle S(t)\cdot S(t+\tau )\rangle }{{\langle S(t)\rangle }^{2}} \end{array}$$

where 〈·〉 indicates the time averaging (we are ignoring the spontaneous emission contribution, small in the parameter range of interest). As is well-known [50], the second-order autocorrelation function gives $g^{\left(2\right)}\left(\tau \right)=1$ for a coherent signal, since its statistics are poissonian. Thermal (or chaotic) radiation, described by Gaussian statistics, gives $g^{\left(2\right)}\left(0\right)=2$, which decays towards $g^{\left(2\right)}\left(\tau \right)\to 1$ as τ→∞: photons that are progressively distant in time become gradually independent; thus, converging towards a Poisson probability distribution.

Superthermal statistics, $g^{\left(2\right)}\left(0\right)>2$, correspond to highly bunched photons, whose mutual, zero-delay (τ=0) correlation is a representation of pulsing behavior [36,42,43,51]. The physical origin of the pulses is the rapid amplification of a fluctuation through stimulated emission due to an excess of accumulated energy in the material (population inversion) [43]. The phenomenon is the extreme form of the depletion that occurs when switching up the pump in a class B laser [52], where coupled oscillations take hold between population and photon number (e.g., spiraling trajectories in phase space [53]). In lasers with a particularly large gain, such as Nd:glass ones [54], the first photon pulse is so large as to deplete the emission to such an extent as to discontinue the stimulated process.

In a nanolaser, the large photon bursts that precede the nanolaser threshold deplete the energy reservoir, causing their own extinction [43]. Since coherence is established and maintained by the action of stimulated emission—even though each photon burst is (partially) coherent with itself (due to the broadband properties of ASE [55,56,57,58]), subsequent pulses are mutually incoherent as they start from a different initiating (spontaneous) photons. Therefore, averaging over a large number of pulses produces radiation with very little coherence. Hence, we can use the information provided by $g^{\left(2\right)}\left(0\right)$ as a first indirect indicator of (in-)coherence in the photon burst regime.

**Figure 1:**
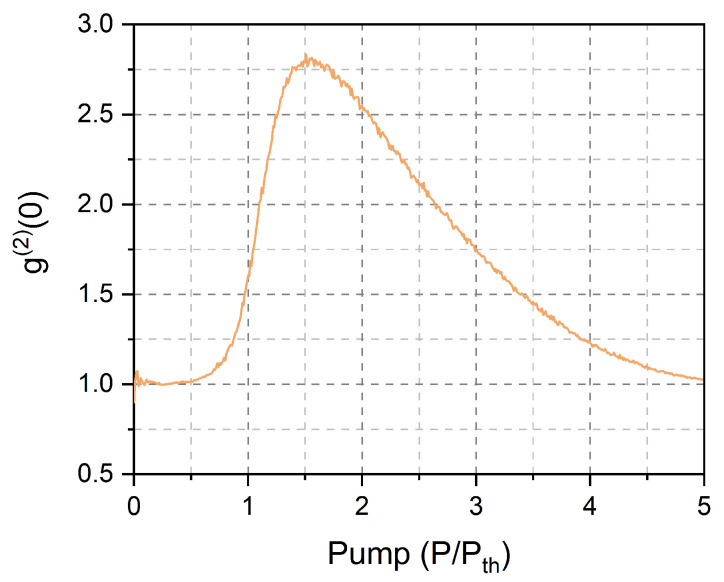
Second-order autocurrelation function ($g^{\left(2\right)}\left(0\right)$) of laser emission with pump.

Figure 1 shows the second-order autocorrelation function of a free-running nanolaser with β=0.1. The autocorrelation exhibits a broad peak with a strongly superthermal maximum [59] at $P\approx 1.5P_{th}$, which slowly decays with a growing pump, not quite reaching full Poissonian statistics (full coherence) in the displayed pump range. We will later show (Section 5.1) that the large autocorrelation value matches the emission of independent pulses, as experimentally observed in a microcavity laser [36].

Superthermal statistics are a guarantee of strong bunching, but the emission of independent photon bursts extends beyond its features. Experimental measurements on a microlaser show that independent photon bursts exist until $g^{\left(2\right)}\left(0\right)\approx 1.6$. Thus, we can infer that low-coherence emissions should exist on a broader pump interval than the one defined by $g^{\left(2\right)}\left(0\right)>2$. Strengthening this case, the experimental measurement of the emission spectrum in a nanolaser, through the first-order autocorrelation $g^{\left(1\right)}$, showed broadband emission in concomitance with superpossonian photon statistics [60]. This feature, normally considered an intrinsic disadvantage of the smallest devices, turns here into an advantage, rendering the realization of low-coherence beams easier than in standard semiconductor lasers [33].

Global considerations predict favorable conditions for illumination applications in a rather broad pump range. At the same time, the lack of phase information in all models grounded in the phenomenological description of the interaction [45], even when fully stochastic [44,61,62,63], recommends particular care in interpreting the predictions. Thus, in the following, we will carefully analyze our results to ensure the plausibility of the predicted low coherence level. Since the latter cannot be quantified within this framework, we propose the challenge for its a posteriori verification in suitable experiments.

## 5. Results and Discussion

We focus our study on the coherence properties of semiconductor nanolasers (using $\beta =10^{-1}$ as a specific example) in the pump range where spontaneous photon bursts appear, with the addition of optical feedback. As we will see in the following, the latter allows for a degree of control of the laser output features, enabling the selection of either larger energy output or of more temporally homogeneous emissions and with better control on the instantaneous power.

### 5.1. Average Emission and Fluctuations in a Free-Running Nanolaser

**Figure 2:**
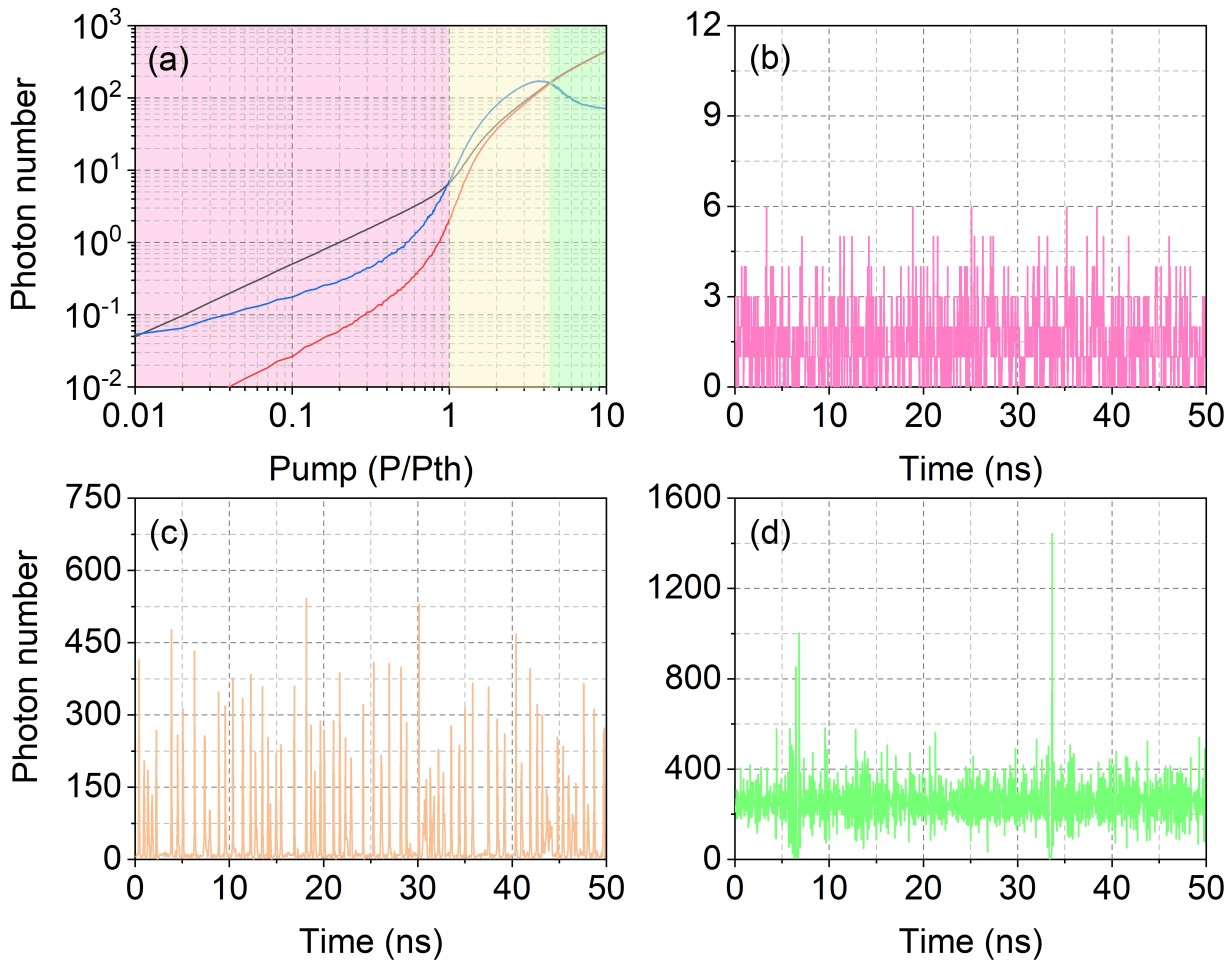
(**a**) Laser output as a function of (normalized) pump: average total emission $\langle L_{T}\rangle$ (black); average stimulated emission $\langle L_{T}\rangle$ (red); standard deviation of the stimulated emission $\sigma_{L_{S}}$ (blue). The yellow region extends from $1\leq \frac{P}{P_{th}}≲4.5$. (**b**–**d**) Temporal dynamics, $L_{T}\left(t\right)$, for different pump values: (**b**) 0.3$P_{th}$; (**c**) $2P_{th}$; and (**d**) $6P_{th}$, respectively.

Figure 2a shows the average total photon output $\langle L_{T}\rangle$ (where $L_{T}=L_{S}+L_{L}$, black curve, with $L_{S}$ and $L_{L}$ stimulated and spontaneous output photons, respectively) and the average stimulated emission $\langle L_{S}\rangle$ (red curve) calculated from the temporal data sequences in a time window $T_{w}=2.5\;\mu$s, shown in logarithmic scale. We are here profiting from the benefit of the stochastic numerical simulation, which allows us to separately observe the spontaneous and stimulated processes to gain insight into the natural constituents of the output—incoherent and coherent photons—a kind of information that is not directly observable in experiments (and typically not even in most traditional laser models). This first piece of information permits quantification of the percentage of the illuminating photons, which are potentially coherent with each other.

The characteristic laser curve, separately displayed for the two components $\langle L_{T}\rangle$ and $\langle L_{S}\rangle$, shows the smooth transition from a dominant spontaneous emission component into a fully coherent output: a feature typical of high-β lasers (irrespective of their construction), leading to the well-known difficulty in identifying the nanolaser threshold [59,64]. As expected, $\langle L_{S}\rangle <\langle L_{T}\rangle$ when $P<P_{th}$, due to the sizeable contribution of the spontaneous emission. This region, especially well below $P_{th}$, resembles the emission characteristics of an LED, whose output is certainly incoherent and can be used for illumination purposes without concern. However, the potential advantages of lasers emerge here. Spontaneous emission is omnidirectional and remains mostly trapped inside the LED. Technological development has enabled stronger emission in a cone that can escape the material, but the collection optics and the transfer of the emission to the sample to be illuminated remains more challenging than with self-collimated laser light. As a result, the laser naturally offers a larger amount of exploitable power—for the same amount of provided energy—without additional efforts; thus, explaining why the oxymoron low-coherence laser light has attracted so much interest [18,19,20,21,23,24,25,26,28,29,30,31,32,33].

At variance with what is known in macroscopic lasers, Figure 2a shows that the nanolaser output does not entirely consist of stimulated photons when $P>P_{th}$. For the β=0.1 (and under the modeling conditions we have chosen) $\langle L_{S}\rangle \approx \langle L_{T}\rangle$ only when $P>4P_{th}$. The actual pump value changes with the chosen parameters and model details, but the first important message to retain is the existence of a broad pump range in which a spontaneous component of the emission remains visible (notice the logarithmic scale of the plot). It is useful to estimate the amount of power emitted in this semi-coherent regime (and around it) by assuming an emission wavelength λ=1μm; thus, converting the photon number in photon energy. At $P=P_{th}$, the conversion gives approximately 100nW of emitted power, growing to the μW range for $P>2P_{th}$. The laser output curve converted into output power, can be found in Section S1 in the Supplementary Material (from here on, all references to sections or figures preceded by “S-” automatically direct the reader to the Supplementary Material). This estimate is useful to have an order of magnitude in the power expected for illumination purposes, as well as for detection (as discussed in Section 2).

The presence of spontaneously emitted photons in the laser output confers a degree of incoherence, but the spontaneous photons alone are not sufficient to provide enough useful power for illumination. A quantification of photon bursts, also limiting the degree of coherence, can be achieved through the calculation of the standard deviation, $\sigma_{L_{S}}$, of the stimulated photons and shown in Figure 2a (blue curve). Its extremely large value (compared to $\langle L_{S}\rangle$) at the lowest computed pump point originates from the rare presence of stimulated photons, which amount to only 40% of the laser output at $P=P_{th}$ (pink background). However, the interesting point is that the standard deviation $\sigma_{L_{S}}>\langle L_{S}\rangle$ well beyond $P=P_{th}$ (region with yellow background). The information coming from the relative size of the standard deviation corresponds to the presence of independent photon bursts. A simple model (Section S2) shows how one can understand that $\rho =\frac{\sigma_{L_{S}}}{\langle L_{S}\rangle }>1$ corresponds to pulses separated by the absence of stimulated emission. Thus, we can consider the yellow region as a good candidate for low-coherence emission, even though from $P>2P_{th}$ more than 90% of the photons stem from a stimulated process. Notice that superthermal statistics (Figure 1) would provide a narrower pump interval in which to expect incoherent emission, confirming our previous remarks.

Figure 2b–d shows examples of computed temporal laser output for three different pump values: below threshold ($P=0.3P_{th}$, panel (b)), in the low-coherence region ($P=2P_{th}$, panel (c)) and for (predominantly) coherent emission ($P=6P_{th}$, panel (d)). A fluctuating photon number characterizes the below-threshold signal (Figure 2b) mostly constituted by spontaneously emitted photons. Twice above $P_{th}$, the output consists of large photon bursts separated by a very low (spontaneous) background: here the peak photon number is more than one order of magnitude larger than its average (Figure 2c). This is the ASE-like regime where low phase coherence arises from the independence of the pulses since each of them grows out of noise; thus, devoid of a correlation with previous ones. Figure 2d shows the shape of the signal emitted when the pump is six times above $P_{th}$: a noisy, continuous output is interrupted by strong fluctuations in the shape of bursts or groups of bursts, whose amplitude can be more than four times the average. However, the (nearly) uninterrupted stream of photons generated by the laser through stimulated emission is an indication of a degree of coherence presumably too large for illumination, even though the substantial amount of photon noise belies what is normally considered good lasing.

### 5.2. Introduction of Optical Feedback

The photon burst region illustrated above also exists in macroscopic lasers—as predicted by stochastic simulations—but the corresponding pump interval is so narrow as to be extremely difficult to find numerically (less than $10^{-4}$ in the normalized units used here) and entirely inaccessible in experiments (well below any reasonable pump stabilization). Thus, the macroscopic burst region, spanning a few units in normalized pumps, represents a regime with properties peculiar to nanodevices. Optical feedback does not render the photon bursts coherent but excites new ones under the action of a fraction of a previous burst reinjected into the cavity [65]. Here, we are going to examine its influence on the dynamics and infer the changes that it imposes on the coherence of the laser output.

**Figure 3:**
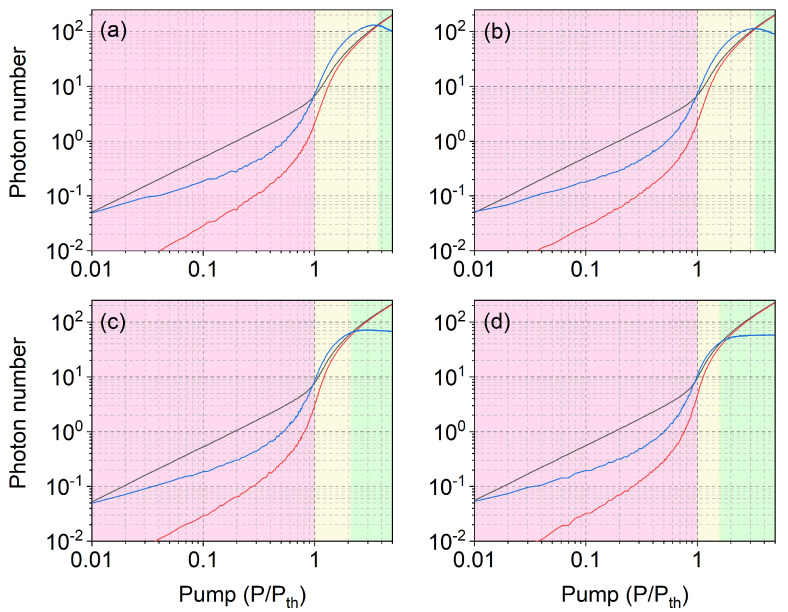
Average total emissions (black) and stimulated fractions (red) in a β=0.1 nanolaser for feedback fraction: 0.5% (**a**); 1% (**b**); 5% (**c**); and 10% (**d**), respectively. The blue curves are the corresponding standard deviations of the stimulated emission.

Figure 3 repeats the information given in Figure 2a, for the free-running regime, in the presence of varying amounts of feedback (the maximum considered is 10%, in order to save most of the output power for illuminating the sample). The progression shows that the photon burst region shrinks with increasing feedback, with the stimulated emission becoming gradually more dominant and its standard deviation more rapidly reducing to the level of the average coherent signal (cf. discussion in Section 5.1). We thus conclude that the coherent fraction is growing, with a corresponding reduction of the fluctuation amplitude. These remarks strongly hint to a transformation of the signal into a coherent output, but, as shown below, this would be an incorrect deduction.

**Figure 4:**
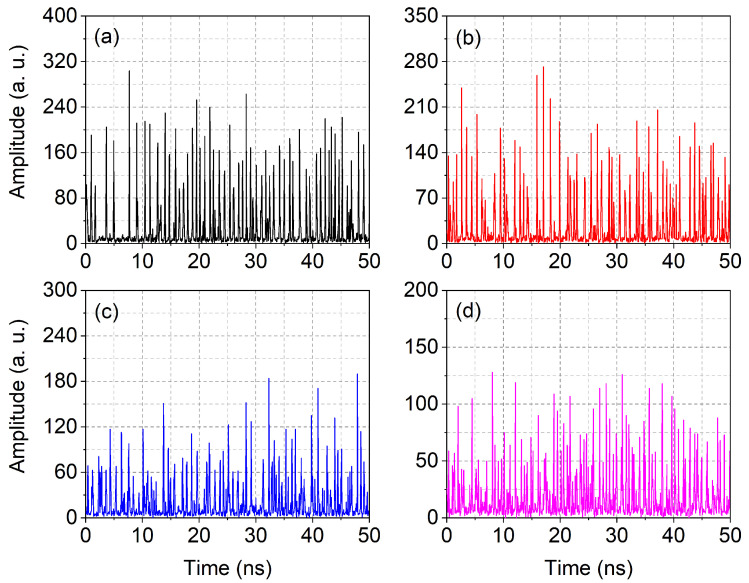
Sample of temporal dynamics obtained under the following conditions: $P=1.6P_{th}$, η=0.005, $\langle L_{T}\rangle \approx 27.1$ (**a**); $P=1.5P_{th}$, η=0.01, $\langle L_{T}\rangle \approx 24.1$ (**b**); $P=1.3P_{th}$, η=0.05, $\langle L_{T}\rangle \approx 19.4$ (**c**); $P=1.15P_{th}$, η=0.1, $\langle L_{T}\rangle \approx 16.4$ (**d**).

Inspection of the temporal dynamics (Figure 4) provides illuminating evidence to interpret the results of the previous figure. Examples of dynamics for different feedback levels are chosen at pump values, for which the relative standard deviation is at a maximum ($\rho_{max}$) (cf. Section S2). The time traces, which display the total emission (i.e., including the spontaneous contribution), clearly show the persistence of sharp photon bursts, mostly interrupted by returns to zero (stimulated emission). Instead, what changes is the amplitude and the frequency with which photon bursts occur: at smaller feedback, the pulses reach a larger photon number but remain less frequent. Thus, the increase in the fraction of the stimulated emission does not come at the expense of the establishment of a continuous stream of photons emitted since there is (almost) always a return to zero (stimulated) photons between subsequent pulses.

The physical change corresponds to a reduction in pulse amplitude in favor of decreased interpulse time delay. Since the population is accumulated in the time intervals with no (or little) emission, the more frequent pulses—stemming from a more efficient reinjection (due to larger feedback)—use the excess population more readily, which, therefore, cannot grow to levels that are as large as the lowest feedback. This way, it is possible to gain a degree of control on the (average) frequency with which light is shone onto the sample and on its granularity (i.e., fluctuations). It is interesting to notice that the average photon number decreases with increasing feedback (cf. figure caption). The figures also show a slight increase in the background (spontaneous emission contribution) for increasing feedback. The decrease in average photon number is not due to a strange effect of feedback but, rather, to the choice of operating the laser at the pump value corresponding to $\rho_{max}$. Since the photon burst region shrinks with increasing feedback, the laser is operated at lower pump values, hence the lower total output.

Finally, the autocorrelation function $g^{\left(2\right)}\left(0\right)$ (cf. Figure S3) confirms that superthermal statistics are found for the conditions of Figure 4a–c and that $g^{\left(2\right)}\left(0\right)\approx 1.8$ for those of Figure 4d (thus above the experimentally observed limit, $g^{\left(2\right)}\left(0\right)≳1.6$, for photon bursts—Section 4). This finding supports the statement of independent pulses, thus enabling the inference of low field coherence.

### 5.3. Possible Feedback-Induced Pulse Interdependence

The question arises naturally, at this point, whether feedback can induce interdependence and to what extent this can influence the coherence of nanolaser output. The radiofrequency spectra of signals taken in the same conditions of Figure 4 provide some information, which can be summarized as follows (cf. Figure S4 and additional information in Section S5): at a low feedback level (η≲0.01), only a minor trace of the frequency stemming from the feedback arm is visible, as well as weak components of the intrinsic frequency associated with the recovery time of the system after the depletion caused by the photon bursts (close to four times the feedback frequency); at a larger feedback level, a very noisy frequency component associated with the action of the feedback arm can be recognized, together with a clear comb of frequencies which are multiples of the “external cavity”, strengthened by the closeness with the intrinsic recovery (inverse) time constant. The comb is most pronounced at η=0.1.

These considerations raise concerns about the possible instauration of coherence through the cyclic renewal of pulses through reinjection. We, therefore, resort to a more detailed analysis of the conditions under which the pulses are generated.

Simultaneously extracting from the numerical code, as separate entities, the number of intracavity stimulated photons and that of reinjected (stimulated) ones, we can perform a cross-correlation analysis to investigate the presence of information transfer from previous into subsequent pulses. We ignore the spontaneous photons for two reasons: 1. their much smaller number renders the likelihood of reinjection negligible; 2. even if a spontaneously emitted photon were to stimulate a pulse, its consequent phase would be randomly distributed with respect to the other pulses. Thus, we only concentrate on the stimulated fraction.

It is important to reiterate the fact that all processes are stochastic, including the reinjection; thus, the feedback percentage is to be interpreted in the sense of a probability and not as a proportionality constant. This point is crucial because there is no deterministic relationship between the returning pulse—and its arrival time—and the actual presence of a reinjected photon inside the cavity: the first reinjected photon may belong to the middle or even the tail of the pulse and, therefore, may arrive, in time, when the intracavity pulse has already developed. Throughout the paper, whenever we speak of a pulse, it is to be intended as one which starts and ends with S=0, i.e., the absence of stimulated photons. Thus, we are certain that there is no interdependence in the coherence properties among pulses.

**Figure 5:**
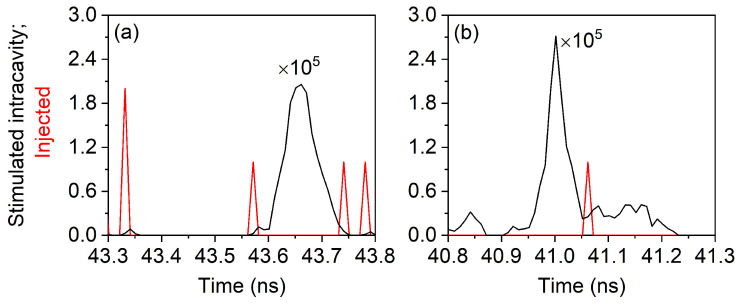
Temporal superposition of intracavity pulse generation (black line) and reinjection of stimulated photons from a previous pulse (red line). (**a**) The injected photon takes place at the beginning of the black pulse (a second one is coupled into the cavity at the end); (**b**) the injected photon intervenes in the middle of the pulse (in time, but late in terms of photon fraction). Notice that the scales for the two kinds of curves differ by five orders of magnitude. This example is taken for the lowest value of η considered; thus, the reinjection consists mostly of one photon at a time. For η=0.1, pulses with up to nine photons are observed (with small likelihood), but their number remains small compared to the intracavity photon number.

The relationship between intracavity pulse emergence and reinjected (stimulated) photons is illustrated in Figure 5, where the left panel shows the reinjected photon (red line) anticipating the intracavity generation process (black line): we can plausibly assume that the reinjected photon may have induced the generation of the large pulse (notice the widely different vertical scales) and may have imposed a phase coherence between the previous pulse (of which the “red” photon is the messenger) and the new pulse. The right panel, instead, shows a situation where one single reinjected photon arrives in the middle of the intracavity pulse (in time) and adds to the already existing intracavity stimulated photons, which outnumber it by more than four orders of magnitude: it is more than plausible that no phase relationship will exist between the black pulse and the preceding one from which the reinjected photon stems. In addition, we see that in this example, the largest part of the photons that make up the black pulse has already been emitted through decay.

One additional point about synchronicity emerges from these pictures. The intracavity pulses have random shapes, which are not maintained during the transmission through the output mirror since the process is stochastic, while free-space propagation maintains the shape of the pulse once it has escaped the cavity, the reinjection, as a stochastic process, further reshapes the reinjected pulse. Hence, no strict timing relationship can be expected in the potential synchronization between intracavity and reinjected pulses. These considerations help to understand the broad peaks in the radiofrequency spectra shown in Section S5.

Finally, η represents the fraction of the output that is fed back towards the laser, but not the actual probability of reintroduction into the cavity, since the output coupler has to be crossed: the actual probability of reinjection is, therefore, much lower.

The computation of the (normalized) cross-correlation uses the standard definition [66,67]:

(3) $$\begin{array}{c} C_{o,f}=\frac{\langle \left[S\left(t\right)-\langle S\rangle \right]\cdot \left[S_{inj}\left(t\right)-\langle S_{inj}\rangle \right]\rangle }{\sqrt{\langle {\left[S\left(t\right)-\langle S\rangle \right]}^{2}\rangle }\cdot \sqrt{\langle {\left[S_{inj}\left(t\right)-\langle S_{inj}\rangle \right]}^{2}\rangle }} \end{array}$$

where the indices *o* and *f* stand for “output” and “feedback”, respectively, and 〈·〉 represents, as usual, the time average. The two terms in the denominator are a shorthand form for the standard deviation of each signal (*S* and $S_{inj}$). As is well-known, this normalized form of the cross correlation provides results in the range $-1<C_{o,f}<1$.

**Figure 6:**
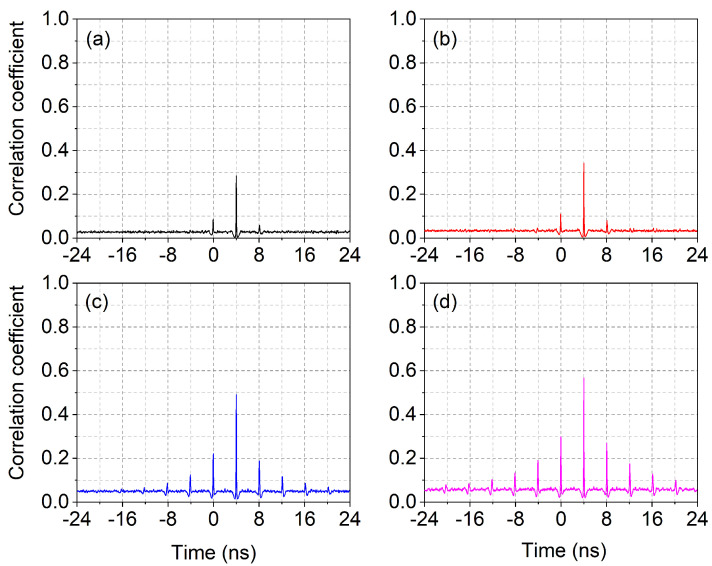
Cross-correlations between intracavity stimulated photons and fraction of reinjected (stimulated) photons from previous pulses. Parameters: $P=1.6P_{th}$, η=0.005 (**a**); $P=1.5P_{th}$, η=0.01 (**b**); $P=1.3P_{th}$, η=0.05 (**c**); $P=1.15P_{th}$, η=0.1 (**d**).

Figure 6 shows the calculated cross-correlation coefficients between the injected and emitted pulses with different feedback fractions. Aside from a low homogeneous offset, the correlation is entirely positive with the main peak placed at the external cavity time $2\tau_{ext}$. Asymmetric lateral peaks appear, with a bias in favor of τ=0, given that the double roundtrip experiences more losses than the zero-delay in the temporal sequence. Starting from η≈0.05, several lateral peaks emerge from the cross-correlation at multiples of the external cavity time, and the central peak grows considerably. This mirrors the behavior of the autocorrelation (Figure S3 and discussion in Section S4), where multiple peaks appear and the statistics barely reach (or dip below) the superthermal regime. At the same time, the radiofrequency spectra (Section S5) also show a multitude of recurrences in the form of a comb of frequencies. It is, therefore, legitimate to speculate on the possible instauration of a regime of mutually coherent pulses mediated by the optical feedback. If this were the case, we could expect a growing degree of coherence, albeit not fully developed, given the persistent irregularity of the temporal spikes (Figure 4). It is worth noticing, in any case, that the autocorrelation, as well as the radiofrequency spectra, show the emergence of a substantial periodicity, while the indications coming from the cross-correlation are much weaker, signaling the possibility that periodicity and coherence may not be strongly interrelated: the resurgences taper off much faster, as visible from the comparison between Figure 6 and Figure S3.

### 5.4. Detailed Pulse Analysis with Optical Feedback

In order to delve a bit deeper into the understanding of the picture, we identify the intracavity photon bursts that coincide with the coupling of an external photon into the cavity. We are starting from the assumption, illustrated by Figure 5, that injected photons that intervene after the generation of the intracavity pulse will not introduce a robust phase relationship. This is quite reasonable, especially observing that the number of reinjected photons is typically at least three orders of magnitude smaller than the number of intracavity stimulated photons: an external signal will stand a chance of introducing phase coherence only if it is the initiator of the pulse. One should also keep in mind the fact that pulses, as mentioned in the introduction for ASE, possess a moderate amount of coherence (typical bandwidths can be a couple of nanometers); thus, one should not expect a strong coherence to emerge.

The results are summarized in Table 1, where the first column gives the percentage of the emitted pulses reinjected into the cavity, and the second one the ratio of the intracavity pulses that do not coincide with a reinjection (“spontaneous”) to the total number of pulses detected in time $T_{w}$. The trend agrees with the previous considerations since for η≲0.01, the majority of pulses is “spontaneously” generated, while above the reverse holds.

**Figure 7:**
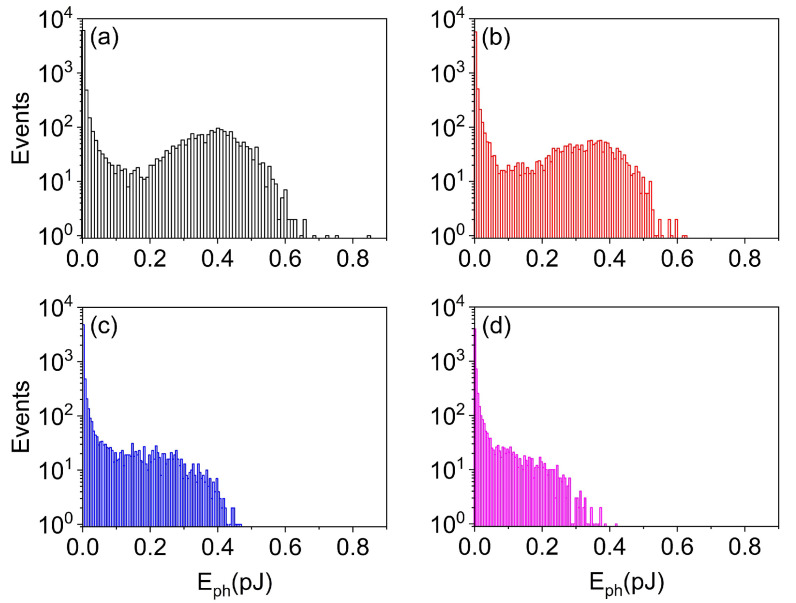
Histogram of energy distribution for feedback fractions η=: 0.005 (**a**); 0.01 (**b**); 0.05 (**c**); and 0.1 (**d**), respectively. Pump: $P=1.6P_{th}$; $P=1.5P_{th}$ (**b**); $P=1.3P_{th}$ (**c**); and $P=1.15P_{th}$ (**d**).

Armed with all the previous considerations, which warn against larger feedback fractions, we perform an additional step in the analysis: we measure the amount of energy contained in each pulse, assuming an emission wavelength λ=1μm. The results are plotted in Figure 7. There is a very clear modification in the statistics of the energy carried by each pulse as a function of feedback: for η≲0.01, the likelihood of more energetic pulses is higher and shows a clear maximum around 0.4 pJ, with the largest pulses as large as 0.8 pJ in the sample we have considered. Instead, for stronger feedback, we not only see more frequent pulses, but their energy distribution progressively becomes a monotonically decreasing function with the highest (and unlikely) energy reaching the typical energy of the peak at low feedback. This result is encouraging since weaker pulses are less likely to bring a strong phase imprint onto following ones, given their smaller photon number, which reduces the mutual influence. We, therefore, see that increasing feedback introduces a (partial) temporal regularization of the nanolaser emission while also reducing the spread in the energy carried by each pulse and introducing a strong bias towards low-energy pulses. This is good news if one desires to have a more regular illumination at short timescales, but also if one wants to avoid pulses that are particularly energetic.

**Figure 8:**
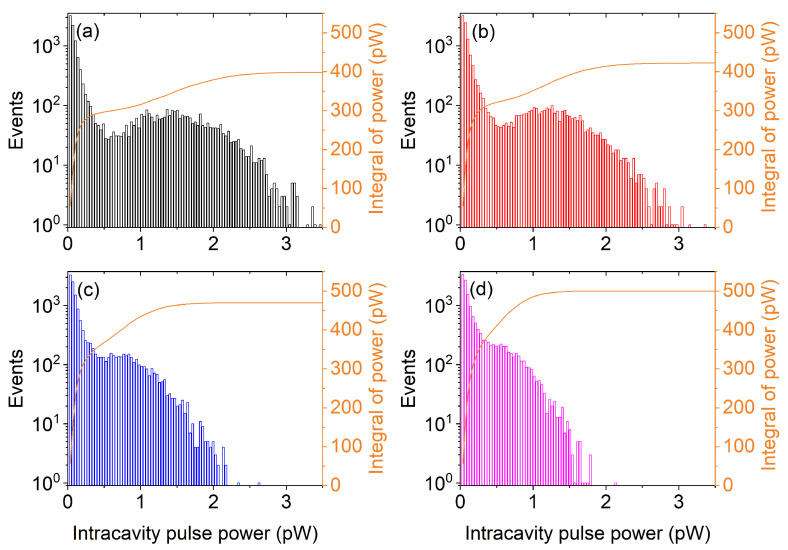
Histograms of pulse power distribution for feedback fractions η=: 0.005 (**a**); 0.01 (**b**); 0.05 (**c**); and 0.1 (**d**), respectively. Pump: $P=1.6P_{th}$; $P=1.5P_{th}$ (**b**); $P=1.3P_{th}$ (**c**); and $P=1.15P_{th}$ (**d**).

The information obtained from Figure 7 presents only the energy side of the question. Figure 5 shows that the pulse shapes can be quite different; thus, conferring a different level of power to each pulse. Making the assumption that the photon number is regularly distributed over its time duration (an assumption that is belied by Figure 5, but which can be used to get some average guidance information), we can obtain the power distribution by dividing the energy of each pulse by its duration. The results are shown in Figure 8. The distributions are even more peaked towards low power values; thus, showing that the power distribution of the illumination source is even more uniform when using larger feedback fractions.

The orange curves show the integral of the histogram; thus, conveying information about the total amount of power carried by the weaker pulses. The largest feedback provides the fastest growth; thus, confirming the comment about the temporal uniformity of the power distribution: the 80% mark is reached, for η=0.1 at a value that is approximately three times lower than for η=0.005.

**Figure 9:**
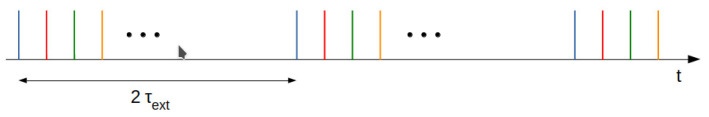
Illustration of the multiple repetitions of pulses in an external cavity roundtrip time $2\tau_{ext}$: the colors indicate independent pulse trains.

The question about possible coherence is still not entirely settled. One piece of information, however, provides precious insight. The last column of Table 1 shows the average number of pulses contained in an external cavity roundtrip time $2\tau_{ext}$. The average is around 20 (somewhat lower for lower feedback, higher for a larger one). This implies the presence of independent trains of pulses, which (irregularly) repeat in a way similar to the pictorial illustration of Figure 9.

The origin of the multiple pulses per external cavity roundtrip is grounded in the internal time constants of the laser at the pump rates that we consider and in the approximate synchronization mentioned above. Since the nanolaser can emit pulses with preferential frequency larger than the external cavity roundtrip, the different trains that appear in the output, measured as the average number of pulses in $2\tau_{ext}$, are independent of one another. Thus, there is no expected mutual phase relationship since the considered trains have been generated during the reinjection time. As a consequence, the amount of coherence, which could be expected from previous indicators—such as the cross-correlation, which does not take into account the origin of the pulses but only counts the “coincidences”—has to be reduced by the number of effective pulse trains. This realization, compounded with the predominance of small pulses at larger feedback values, lends confidence to the possibility of obtaining a low coherence output even from larger feedback.

The fact that multiple trains of pulses can exist in a cavity roundtrip time suggests checking whether the observation still holds for longer feedback arms. Considering a roundtrip time $2\tau_{ext}=10$ ns and η=0.1, we obtain results that are extremely similar to those at a shorter delay time, the main difference being that the average number of pulses passes from ≈24 to ≈57, i.e., a multiplicative factor nearly equal to the ratio between the feedback arms. This implies that the amount of residual coherence could be controlled in this way, with longer feedback arms providing a better nanosource for illumination. The arm length for 10 ns delay amounts to 1 m if an optical fiber is employed: a reasonable length that ensures good coupling and sufficient stability. The main difference will be a longer initial transient, from the switch-on of the source until the pulses have settled in a statistically stable configuration. As already mentioned, most of our simulations have been done with a shorter delay to keep the computing time down to more reasonable values.

## 6. Conclusions

The presence of “unusual”—for macroscopic lasers—spontaneous pulsing offers the possibility of obtaining low-coherence directional emission from a nanolaser. The great advantage is that this kind of emission is naturally present below the threshold and does not require any engineering effort. The features of this emission can be, however, tailored to the users’ needs by introducing a small amount of optical feedback. The analysis has shown that the latter can regularize the emission in time by introducing smaller and more frequent pulses organized in independent trains whose number depends on the length of the feedback arm. The latter can be constructed by coupling the output into an optical fiber of easily handled length (of the order of one meter) to substantially modify the power distribution.

The current analysis has been based on a fully stochastic simulation of the laser operation, an important feature, since it has allowed for the proper simulation of the probabilistic nature of the transmission (in output and in reinjection) both concerning the photon number (thus the pulse shape) and the time at which the event takes place. As discussed in Section 3, the unavoidable shortcoming of this approach is the lack of direct information on the linewidth—thus coherence. These promising forecasts now need to be tested experimentally to answer whether nanolasers can be truly used as low-coherence sources for speckle-free (or speckle-reduced) illumination. 

## Figures and Tables

**Table 1:** Percentage of spontaneous and stimulated pulses within total pulses.

Feedback Fraction	Ratio Spontaneous/All Pulses	Independent Pulse Trains
0.5%	0.76	18.2
1%	0.67	19.3
5%	0.35	21.5
10%	0.24	22.9

## Data Availability

Data will be made available upon reasonable request.

## Supplementary Materials

The following are available online at https://www.mdpi.com/article/10.3390/nano1010000/s1, Figure S1: (a) Output photon number as a function of pump (black); power output by the nanolaser (red). (b) Second-order autocorrelation function ($g^{\left(2\right)}\left(0\right)$) as function of pump. β=0.1. Figure S2: Calculated values of ρ (“Ratio” in vertical scale) for η= 0.005 (a); 0.01 (b); 0.05 (c); and 0.1 (d). Figure S3: Second-order autocorrelation function ($g^{\left(2\right)}\left(\tau \right)$) with time delay for the dynamics observed at: $P=1.6P_{th}$ and η=0.005 (a), $P=1.5P_{th}$ and η=0.01 (b), $P=1.3P_{th}$ and η=0.05 (c), and $P=1.15P_{th}$ and η=0.1 (d). Figure S4: Nanolaser rf spectra for the temporal dynamics corresponding to:$P=1.6P_{th}$ and η=0.005 (a), $P=1.5P_{th}$ and η=0.01 (b), $P=1.3P_{th}$ and η=0.05 (c), and $P=1.15P_{th}$ and η=0.1 (d).
