# Supplementary material for: Nanolasers with Feedback as Low-Coherence Illumination Sources for Speckle-Free Imaging: A Numerical Analysis of the Superthermal Emission Regime
Source: Nanomaterials (Basel). 2021 Dec 7;11(12):3325. doi: 10.3390/nano11123325 (PMC8708746; doi:10.3390/nano11123325)
Supplement: Supplementary file 1 [file nanomaterials-11-03325-s001.zip › nanomaterials-1473451-supplementary.pdf]

nanomaterials

Supplementary Materials

Nanolasers with Feedback as Low-Coherence Illumination Sources for Speckle-Free Imaging: A Numerical Analysis of the Superthermal Emission Regime

Tao Wang [1,*], Can Jiang [1], Junlong Zou [2], Jie Yang [1], Kuiwen Xu [1], Chaoyuan Jin [3,4,5], Gaofeng Wang [1,*] ,Gian Piero Puccioni [6] and Gian Luca Lippi [7,*]

1.School of Electronics and Information, Hangzhou Dianzi University, Hangzhou 310018, China; jiangcan@hdu.edu.cn (C.J.); yanglime@hdu.edu.cn (J.Y.); kuiwenxu@hdu.edu.cn (K.X.)
2 School of Communication Engineering, Hangzhou Dianzi University, Hangzhou 310018, China; zjl418597114@hdu.edu.cn
3 College of Information Science and Electronic Engineering, Zhejiang University, Hangzhou 310007, China; jincy@zju.edu.cn
4 International Joint Innovation Center, Zhejiang University, Haining 314400, China
5 Zhejiang Lab, Hangzhou 311121, China
6 Istituto dei Sistemi Complessi, CNR, 50019 Sesto Fiorentino, Italy; gianpiero.puccioni@isc.cnr.it
7 Institut de Physique de Nice (INPHYNI), Universite Cote d'Azur, 06103 Nice, France
* Correspondence: wangtao@hdu.edu.cn (T.W.); gaofeng@hdu.edu.cn (G.W.); gian-luca.lippi@inphyni.cnrs.fr (G.L.L.)

Section S1. Nanolaser output power and zero-delay second-order autocorrelation

The black curve in Figure S1a shows the output photon number of a nanolaser with β = 0.1 (other parameters given in the main paper) as a function of (normalized) pump. If we suppose that the emission wavelength is $\lambda = 1\mu$m, we can compute the output power (red curve and red scale on the right-hand-side of the graph). The power can reach 4 μW for pump $P = 5P_{th}$. Figure S1b shows the second-order zero-delay autocorrelation function of the output photons ($g^{(2)}(0)$) as a function of pump. $g^{(2)}(0)$ quickly grows beyond the conventionally fixed "threshold" value [1] (not matching the real threshold in a nanolaser), reaching a maximum at $P = 1.5P_{th}$. The ensuing decay is slow and the autocorrelation does not reach fully coherent emission ($g^{(2)}(0) = 1$) even at $P = 5P_{th}$. The functional shape of $g^{(2)}(0)$ indicates a broad transition between fully spontaneous and laser emission. We remark that in the interval $1.1 \lesssim \frac{P}{P_{th}} \lesssim 2.7 P_{th}$ the autocorrelation $g^{(2)}(0) > 2$ indicating superthermal emission statistics.

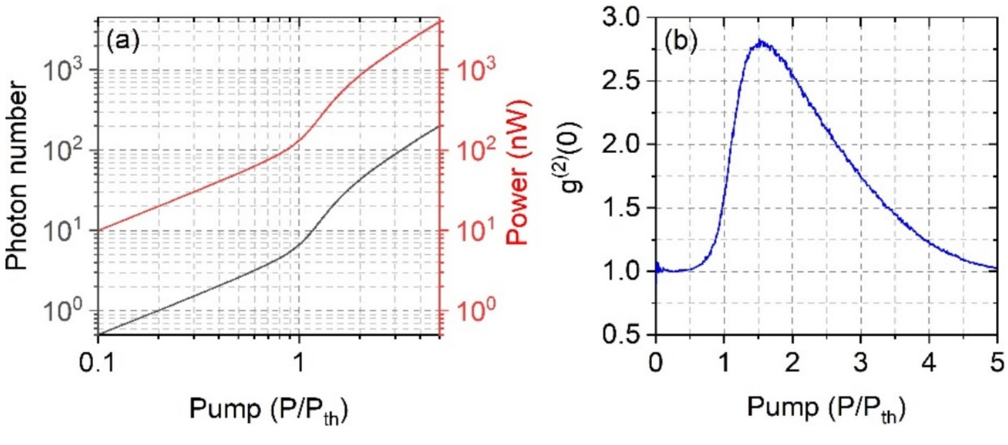

Figure S1. (**a**) Output photon number as a function of pump (black); power output by the nanolaser (red). (**b**) Second-order autocorrelation function ($g^{(2)}(0)$) as function of pump. β = 0.1.

Section S2. Estimate of the conditions on the relative standard deviation

One of the indicators used in the main paper to assess the presence of photon bursts is the relative amplitude of the signal's standard deviation compared to its average. In the general case, the complex sequence of irregularly shaped and spaced pulses, of widely different amplitude (over orders of magnitude), which constitute the laser output is difficult to analyse. However, we can get an idea of the conditions under which the standard deviation of the signal can exceed its average on the basis of a simple example.

Suppose a square signal *s(t)* of amplitude *A*, over a 0 offset, period* *T* and duty cycle δ:

* Periodicity is not indispensable, since the signal can have a single peak in the measurement time *T*. It renders, however, the rest of the reasoning simpler.

$$s(t) = \begin{cases} A, & 0 \le t \le T\delta \\ 0, & T\delta \le t \le T \end{cases} \tag{S1}$$

Sampling with a time step τ, supposed commensurate with δ and *T*, provides an ensemble of *N* measured points ($N_\tau = T$), from which the average is computed in a straightforward way:

$$\langle S \rangle = \frac{N_\tau A\delta}{N_\tau}, \tag{S2}$$

$$= A\delta \ , \tag{S3}$$

since computation over the time interval *T* suffices for a periodic signal. The same argument applies for the next step.

Its variance, defined for an ensemble of *M* samples s_j as

$$\sigma^2 = \frac{\sum_{j=1}^{M}(s_j - \langle s \rangle)^2}{M-1} \tag{S4}$$

$$\sigma^2 = \frac{N_\tau \delta (A - A\delta)^2 + N_\tau (1-\delta)(A\delta)^2}{(N-1)\tau} \tag{S5}$$

$$\approx A^2[\delta(1-\delta)^2 + (1-\delta)\delta^2] \tag{S6}$$

$$= A^2[\delta - \delta^2] \tag{S7}$$

where, in passing from eq. (S5) to eq. (S6), we have supposed a sufficiently large number of measurement points ($N \gtrsim 10$).

Computing the ratio between the variance and the square of the average and imposing

$$\frac{\sigma}{\langle s \rangle} > 1 \tag{S8}$$

We immediately obtain

$$\frac{\sigma^2}{\langle s \rangle^2} = \frac{\delta - \delta^2}{\delta^2} \tag{S9}$$

$$\delta - \delta^2 > \delta^2 \tag{S10}$$

$$\delta < \frac{\sqrt{2}}{2} \tag{S11}$$

Thus, the only condition to be fulfilled (irrespective of the amplitude A) is that the pulse should not occupy more than approximately 0.7*T*. The condition obtained here is quite simple, thanks to the chosen signal.

In the real situation, the expression will be more complex and will depend on the different amplitudes, pulse widths and shapes. An equivalent condition will not be as simple as the one of eq. (S11) and will, in general, need to be solved numerically. However, the principle stands and its simple illustration helps understanding the reason why the standard deviation will be larger than the average in a signal which consists of isolated pulses. Since the independence of the pulses, i.e. their being separated by intervals with 0 stimulated photons, is the key to a broader linewidth - thus low coherence -, then the criterion employed in the paper is useful and holds.

Section S3. Functional dependence of the relative fluctuations

In order to delimit the range in which the relative fluctuations indicate pulse independence, we define the relative standard deviation of the signal as $\rho = \frac{\sigma}{\langle s \rangle}$, with the notation of the previous section. The condition expressed by eq. (S8) defines the extrema of region 2 (yellow) depicted in Figure 3 and 4 of the main paper.

Figure S2 displays the dependence of ρ on pump for the four feedback cases discussed in the main paper; the yellow-shaded region is defined by eq. (S8) and its information is used to delimit its boundaries (normalized pump values) in the main paper.

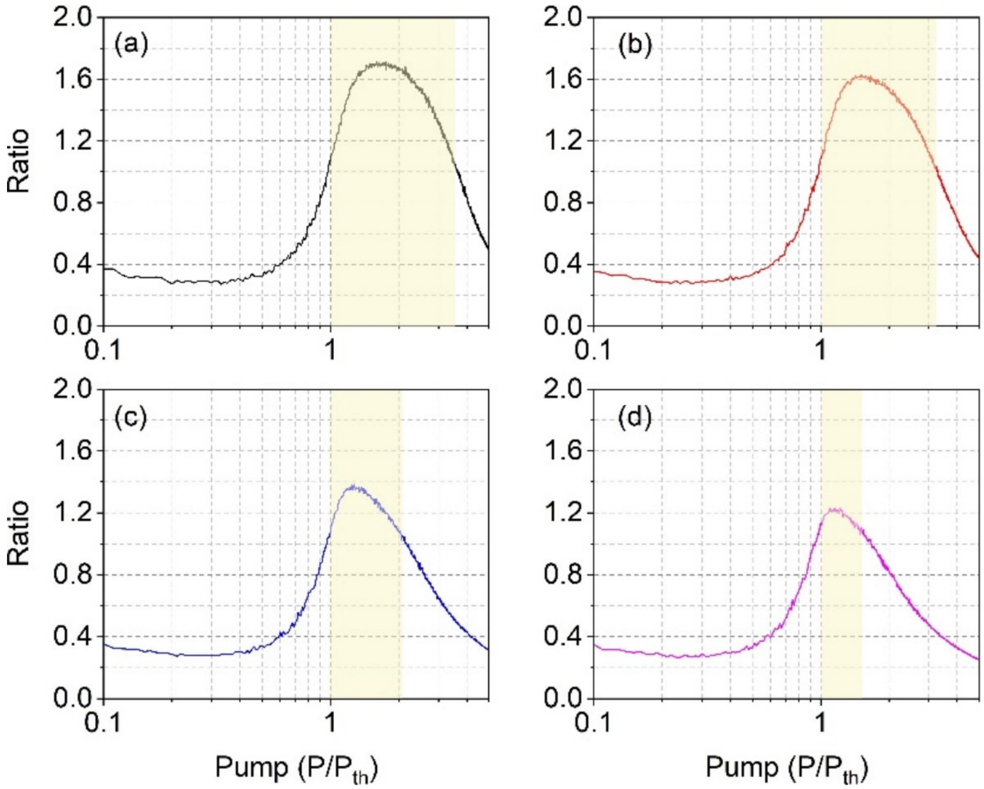

Figure S2. Calculated values of ρ (“Ratio” in vertical scale) for η = 0.005 (**a**); 0.01 (**b**); 0.05 (**c**); and 0.1 (**d**).

Section S4. Time-delayed second-order autocorrelation with optical feedback

Figure S3 shows the temporal second-order correlation functions ($g^{(2)}(\tau)$) for the corresponding dynamics. From this figure, it is possible to read off the graph the value of the zero-delay autocorrelation, used in the discussion of Section 4.2 (main paper).

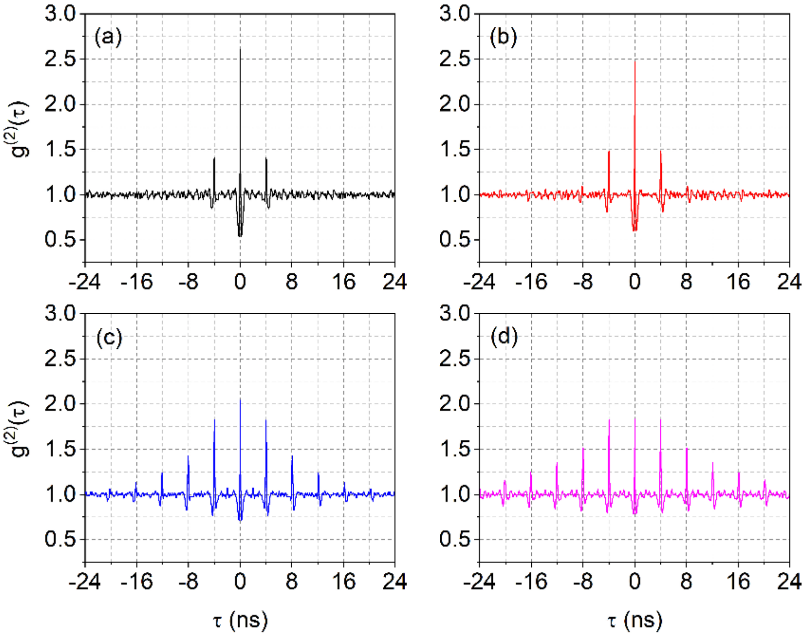

Figure S3. Second-order autocorrelation function ($g^{(2)}(\tau)$) with time delay for the dynamics observed at: $P = 1.6P_{th}$ and $\eta = 0.005$ (**a**), $P = 1.5P_{th}$ and $\eta = 0.01$ (**b**), $P = 1.3P_{th}$ and $\eta = 0.05$ (**c**), and $P = 1.15P_{th}$ and $\eta = 0.1$ (**d**).

When the feedback fraction is very weak, ~0.5%, a large central peak (at zero delay) and two side peaks are observed (Figure S3a). The central peak associated with $g^{(2)}(\tau) > 2.5$ identifies superthermal photon statistics associated with isolated pulses, while the two small side-peaks identify a residual autocorrelation at the level of the external cavity roundtrip. Increasing the feedback fraction to 1% sees a reduction in the superthermal component, accompanied by a growth of the autocorrelation with the returning pulses. A further increase, to 5%, enhances the growth of the side-peaks with the autocorrelation extending to four cavity roundrips and barely superthermal statistics at $\tau = 0$. Finally, when the feedback fraction reaches 10%, $g^{(2)}(\tau) < 2$ signals the end of superthermal statistics; however, we still find $g^{(2)}(0) > 1.6$ (cf. main paper). It is interesting to note that at this feedback value the autocorrelation with the first cavity roundtrip is as large as the one at $\tau = 0$ and the decay with additional roundtrips is slower.

S5. Radiofrequency spectra of the signal with optical feedback

Fourier-transforming the temporal signals computed at ρ_{max} for the different feedback levels we obtain the radiofrequency (rf) spectra shown in Figure S4, displayed in double-logarithmic scale. At the lowest feedback level (panel (a)) the rf spectrum possesses almost no structure: one can barely recognize a small feature around 1 GHz, matching the nanolaser internal time constants. This peak become slightly more noticeable, together with a few harmonics, in panel (b), but the overall spectrum remains dominated by noise. Starting from panel (c) a broad component associated with the external cavity (at 0.25 GHz for the values taken in the main paper) starts to emerge [2], becoming more visible in its harmonics and combining with the internal nanolaser's time constants. The periodicity now appears quite clearly, although it wasn't identifiable in the temporal

signal [3]. The features develop even further in panel (d), where a substantial frequency comb can be recognized on top of a noise floor which is at best approximately 20dB below. In spite of the visibility of the different frequency components, the rf spectrum remains quite noisy.

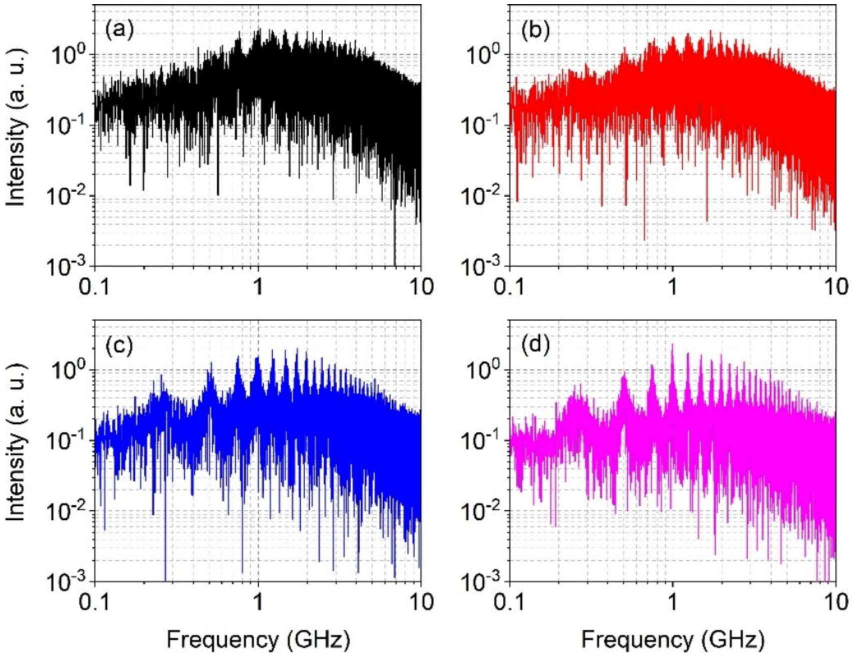

Figure S4. Nanolaser rf spectra for the temporal dynamics corresponding to: $P = 1.6P_{th}$ and $\eta = 0.005$ (**a**), $P = 1.5P_{th}$ and $\eta = 0.01$ (**b**), $P = 1.3P_{th}$ and $\eta = 0.05$ (**c**), and $P = 1.15P_{th}$ and $\eta = 0.1$ (**d**).

References

[1] P. R. Rice and H. J. Carmichael, "Photon statistics of a cavity-QED laser: A comment on the laser–phase-transition analogy," *Phys. Rev. A* **1994**, *50*, 4318.

[2] O. D' Huys, T. Jüngling, and W. Kinzel, "Stochastic switching in delay-coupled oscillators," *Phys. Rev. E* **2014**, *90*, 032918.

[3] T. Wang, Z. L. Deng, J. C. Sun, X. H. Wang, G. P. Puccioni, G. F. Wang, and G. L. Lippi, "Photon statistics and dynamics of nanolasers subject to intensity feedback," *Phys. Rev. A* **2020**, *101*, 023803.
